# Metagenomic Detection and Genetic Characterization of Human Sapoviruses among Children with Acute Flaccid Paralysis in Nigeria

**DOI:** 10.3390/pathogens13030264

**Published:** 2024-03-19

**Authors:** Uwem Etop George, Temitope O. C. Faleye, Lander De Coninck, Sheriff Tunde Agbaje, Ijeoma Maryjoy Ifeorah, Bernard Anyebe Onoja, Elijah Igbekele Oni, Adebowale Oluseyi Olayinka, Toluwani Goodnews Ajileye, Arthur Obinna Oragwa, Toluwanimi Emmanuel Akinleye, Bolutife Olubukola Popoola, Oluwadamilola Gideon Osasona, Olaitan Titilola Olayinka, Oluwadamilola Adefunke George, Ahmed Iluoreh Muhammad, Isaac Komolafe, Adekunle Johnson Adeniji, Jelle Matthijnssens, Moses Olubusuyi Adewumi

**Affiliations:** 1 African Centre of Excellence for Genomics of Infectious Diseases, Redeemer’s University, Ede 232101, Nigeria; george27@run.edu.ng; 2 Department of Biological Sciences, Faculty of Natural Sciences, Redeemer’s University, Ede 232101, Nigeria; toshokomolafe@gmail.com; 3 Center for Environmental Health Engineering, Biodesign Institute, Arizona State University, Tempe, AZ 85281, USA; 4 Laboratory of Viral Metagenomics, Department of Microbiology, Immunology and Transplantation, Rega Institute, Universiteit Leuven, 3000 Leuven, Belgium; lander.deconinck@kuleuven.be; 5 Department of Virology, College of Medicine, University of Ibadan, Ibadan 200212, Nigeria; sheffydeen1@gmail.com (S.T.A.); bernardonoja@yahoo.com (B.A.O.); oni.elijah002@gmail.com (E.I.O.); debowaley2k@gmail.com (A.O.O.); ajileye017@yahoo.com (T.G.A.); bolupopson@yahoo.com (B.O.P.); muhammadahmedilu@gmail.com (A.I.M.); adek1808@yahoo.com (A.J.A.); 6 Department of Medical Laboratory Science, Faculty of Health Science and Technology, College of Medicine, University of Nigeria Enugu Campus, Enugu 400241, Nigeria; ijeoma.ifeorah@unn.edu.ng; 7 Centre for Translation and Implementation Research, University of Nigeria, Nsukka 410001, Nigeria; 8 Department of Veterinary Microbiology, Faculty of Veterinary Medicine, University of Jos, Jos 930003, Nigeria; oragwaa@unijos.edu.ng; 9 Phytomedicine Unit, Department of Pharmacognosy, Faculty of Pharmacy, University of Ibadan, Ibadan 200005, Nigeria; toluwanimiemmanuel@rocketmail.com; 10 Department of Medical Laboratory Sciences, Faculty of Basic Medical Sciences, Redeemer’s University, Ede 232101, Nigeria; osasona23@run.edu.ng; 11 Hospitals Management Board, Ado-Ekiti 360102, Nigeria; 12 National Polio Laboratory, College of Medicine, University of Ibadan, Ibadan 200212, Nigeria; olaitanayinnde@gmail.com; 13 National Veterinary Research Institute, Vom 930101, Nigeria; drdammy@gmail.com; 14 Infectious Disease Institute, College of Medicine, University of Ibadan, Ibadan 200212, Nigeria

**Keywords:** metagenomics, sapovirus, Acute Flaccid Paralysis, Acute Gastroenteritis, Nigeria

## Abstract

Using a metagenomic sequencing approach on stool samples from children with Acute Flaccid Paralysis (AFP), we describe the genetic diversity of Sapoviruses (SaVs) in children in Nigeria. We identified six complete genome sequences and two partial genome sequences. Several SaV genogroups and genotypes were detected, including GII (GII.4 and GII.8), GIV (GIV.1), and GI (GI.2 and GI.7). To our knowledge, this is the first description of SaV infections and complete genomes from Nigeria. Pairwise identity and phylogenetic analysis showed that the Nigerian SaVs were related to previously documented gastroenteritis outbreaks with associated strains from China and Japan. Minor variations in the functional motifs of the nonstructural proteins NS3 and NS5 were seen in the Nigerian strains. To adequately understand the effect of such amino acid changes, a better understanding of the biological function of these proteins is vital. The identification of distinct SaVs reinforces the need for robust surveillance in acute gastroenteritis (AGE) and non-AGE cohorts to better understand SaVs genotype diversity, evolution, and its role in disease burden in Nigeria. Future studies in different populations are, therefore, recommended.

## 1. Introduction

Sapovirus (SaV) infections are a significant public health problem with the virus implicated in acute gastroenteritis (AGE) in humans and animals [1]. The virus has been associated with both outbreaks and isolated cases of AGE among children and adults [2,3,4,5,6,7,8,9,10]. Sapovirus infections frequently cause diarrhea and vomiting, which usually last for about a week [11]. However, people exhibiting symptoms for longer than usual and with greater severity have also been documented, particularly in immune-compromised individuals [12,13]. The asymptomatic circulation of SaVs has also been reported in children without symptoms of AGE [14,15].

The species *Sapporo virus* belongs to the genus *Sapovirus*, family *Caliciviridae.* Sapoviruses of human origin were first discovered in the stools of children with gastroenteritis in 1976 and in fecal samples collected from babies during a stool survey of Glasgow children using electron microscopy (EM). However, the strain Hu/SaV/Sapporo/1982/JPN (thought to have originated from an outbreak in Sapporo, Japan, in 1982) is widely regarded as the Sapovirus genus prototype strain due to its extensive genetic and virologic characterization [1,16]. The virus is non-enveloped, with a positive-sense, single-stranded RNA genome, which is approximately 7.1 to 7.7 kb in length, containing two open reading frames (ORFs). The large ORF1 encodes a polyprotein which is cleaved by a virus-encoded protease into nonstructural proteins (NS1 [p11], NS2 [p28], NS3 [NTpase], NS4 [p32], NS5 [viral genome-linked protein-VPg], and NS6-NS7 [protease–polymerase, which is further cleaved to form an RNA-dependent RNA-polymerase-RdRp]) and VP1 (the major structural protein). The ORF 2 encodes VP2 (the minor structural protein) [17,18,19,20]. A third ORF has been reported, although its function is currently unknown [1,18].

The most variable (both genetically and antigenically) region of SaVs is the VP1 domain, which is important for eliciting immune responses. The classification of SaV is based on complete VP1 amino acid (aa) sequences, with strains exhibiting ≥57% pairwise VP1 aa identity placed in the same genogroup [21]. Currently, SaVs have been classified into 19 genogroups (GI to GXIX), with four genogroups (GI, GII, GIV, and GV) known to infect humans [22,23,24]. SaVs in other genogroups have been identified in minks (GXII), bats (GXIV, GXVI-GXIX), dogs (GXIII), rodents (GXV), swine (GIII and GV-GXI), and sea lions (GV) [25,26,27]. Human SaVs are further classified into 17 different genotypes [1], and a recently detected genotype in Peru has been proposed as GII.8 [11,28].

Metagenomics, an alternate culture- and sequence-independent method, does not require the presence of a specific gene in all subject entities. The original goal for developing this methodology was to enable the sequence-based and functional analysis of collective microbial genomes in environmental samples [29,30]. Viral metagenomics has proven to be an effective technique for discovering new viruses and expanding our understanding of the diversity of viruses found in clinical samples, including the identification of new SaV strains [29,31]. Metagenomic analyses using whole genome sequencing are becoming more common in clinical settings, and they have been used for the in-depth genomic analysis of SaVs in four different countries in the Americas [32] and China [33,34]. The use of whole genome rather than short genome sequences has improved the in-depth analysis of viral genomes, including members in the *Caliciviridae* family that have the capability to rapidly evolve, recombine, and acquire mutations [32,35]. Moreover, metagenomic shotgun sequencing has enabled researchers to track viral infection transmission and conduct effective epidemiological studies. These advancements have contributed to reducing the burden of treatment for patients by preventing and controlling infections [34].

Different genomic regions, particularly those encoding RdRp and VP1, can cause discrepancies in phylogenetic clustering, resulting in the discovery of intra- and inter-genogroup recombinant strains [36]. Similar to noroviruses, several recombinant SaV strains have been reported [36,37,38]. These strains may have changed virulence as a result of recombination, which, in turn, may enhance and increase disease burden [12]. Recombinant SaV strains have been classified as those with a discordant clustering of the VP1 encoding region and the RNA-dependent RNA-polymerase (RdRp) [37], with the RdRp-VP1 junction and the NS3-NS4 junction found to be the two main recombination hotspots [35,36,38].

In Africa, the landscape of circulating human SaV genogroups in recent years has been dominated by GI and GII SaVs [39,40]. Genogroup V (GV) viruses have been rarely reported in Africa. In contrast, GIV has been reported in Burkina Faso [41] and South Africa [42], where they were identified in up to one-third of infections in patients with gastroenteritis [42,43]. There are currently no published data on SaV infections in Nigeria. In this study, we describe the molecular characterization and genetic diversity of SaV genomes identified in the stool samples of children 15 years and below diagnosed with Acute Flaccid Paralysis (AFP) in Nigeria.

## 2. Methodology

### 2.1. Faecal Specimen Collection and Processing

The fecal samples analyzed in this study were collected as part of the National AFP surveillance program in Nigeria. Samples were collected from children aged 15 years and below diagnosed with AFP in Nigeria in 2020 [44]. These stool samples were collected between January and December 2020 following national ethical guidelines and sent to the WHO National Polio Laboratory in Ibadan, Nigeria.

In this study, 254 archived (−20 °C freezers stored) poliovirus culture-negative samples from five states in Nigeria (Supplementary Figure S1) were combined into 55 pools by the state of collection and the month of sample collection and subsequently analyzed. Briefly, about 0.5 g of stool was dissolved in 4.5 mL of phosphate-buffered saline (PBS) and 0.5 g of glass beads. After 20 min of vortexing, the mixture was subjected to 20 min of centrifugation at 3000 rpm. Subsequently, 2 mL of the supernatant was aliquoted into 1 mL cryovials and stored at −20 °C. Thereafter, the stool suspensions were pooled. To make a pool, 200 uL of fecal suspensions were mixed, with each sample pool containing between 1 and 7 fecal suspensions (Supplementary Table S1). Sample pools were subsequently shipped on ice packs to the University of Leuven, Rega Institute, Laboratory of Clinical and Epidemiological Virology in Belgium. The samples were stored at −80 °C until further processing.

### 2.2. Sequencing and Read Processing

The NetoVIR protocol was used to purify virus-like particles (VLPs) from the samples, as previously described [45]. Briefly, using a MINILYS homogenizer, fecal suspensions were homogenized for 1 min at 3000 rpm and filtered through a 0.8 μm PES filter. Free-floating nucleic acids were digested via treatment with a mixture of Benzonase (Millipore, Billerica, MA, USA), (Novagen, Madison, WI, USA), and Micrococcal Nuclease (New England Biolabs, Ipswich, MA, USA). Subsequently, nucleic acid was extracted using the QIAamp Viral RNA Mini Kit (Qiagen, Hilden, Germany) according to the manufacturer’s instructions, but without the addition of carrier RNA. A slightly modified Whole Transcriptome Amplification (WTA2) Kit procedure (Sigma-Aldrich, St Louis, MO, USA) was used for the first- and second-strand synthesis, as well as a random PCR amplification over 17 cycles. The WTA2 products were purified using MSB Spin PCRapace spin columns (Stratec Biomedical, Birkenfeld, Germany). The libraries for Illumina sequencing were prepared using the Nextera XT Library Preparation Kit (Illumina, San Diego, CA, USA). After that, samples were paired-end-sequenced (2 × 150 bp) on an Illumina Novaseq 6000 platform.

Raw reads were processed with the Virome Paired-End Reads (ViPER) pipeline (https://github.com/Matthijnssenslab/ViPER, accessed on 14 March 2024). Using Trimmomatic, the reads were trimmed for quality and adapters [46], and reads mapping to the human genome were removed using Bowtie 2 [47]. Subsequently, the trimmed and filtered reads were de novo assembled into contigs using metaSPAdes [48]. The sensitive option in DIAMOND was then used to annotate the contigs [49]. Kronatool files were manually inspected to identify all SaV genomes. To determine the depth of coverage, trimmed reads were mapped against the SaV contigs using Bowtie2 [40].

### 2.3. Sapovirus Genotyping and Phylogenetic Analyses

A BLASTn search was performed against the GenBank database using the SaV contigs identified in this study as queries. The top five hits (sequences with the highest percentage of identity and query coverage) were downloaded and added to the alignment, along with reference human SaV sequences. The SaV sequences generated in this study were aligned with reference human SaV sequences downloaded from GenBank using the MAFFT online tool [50]. The human calicivirus genotyping tool [51] was used to determine the genogroups and genotypes of each SaV sequence generated in this study. To construct the corresponding maximum-likelihood phylogenetic trees, various genomic regions of interest, including individual genes encoding structural (VP1 and VP2) and nonstructural (NS1-7) proteins, were selected from the alignment. Phylogenetic trees were constructed using MEGA version 11 [52] and the maximum-likelihood method with 1000 bootstrap replications. Subsequently, we aligned each distinct pair of sequences to determine the pairwise identity of the sequences from this study and published reference sequences using the Sequence Demarcation Tool [53]. The conserved amino acid motifs for SaV were identified and analyzed using NCBI’s conserved domain database (CDD) [54]. Sequences from this study were also analyzed for recombination events using the Recombination Detection Program (RDP) 4 [55]. To detect recombination, nine different detection techniques—including RDP, GENECONV, BootScan, MaxChi, Chimaera, 3Seq, PhylPro, LARD, and SiScan—were used with the default parameters. Recombination events were considered reliable if they were predicted by at least six different detection methods in the RDP4 program.

### 2.4. GenBank Submission

The Sapovirus nucleotide sequences and mapped reads described in this study were submitted to GenBank and the SRA and assigned accession numbers OR837774-OR837781 and PRJNA1043841, respectively.

## 3. Results

**Figure 1:**
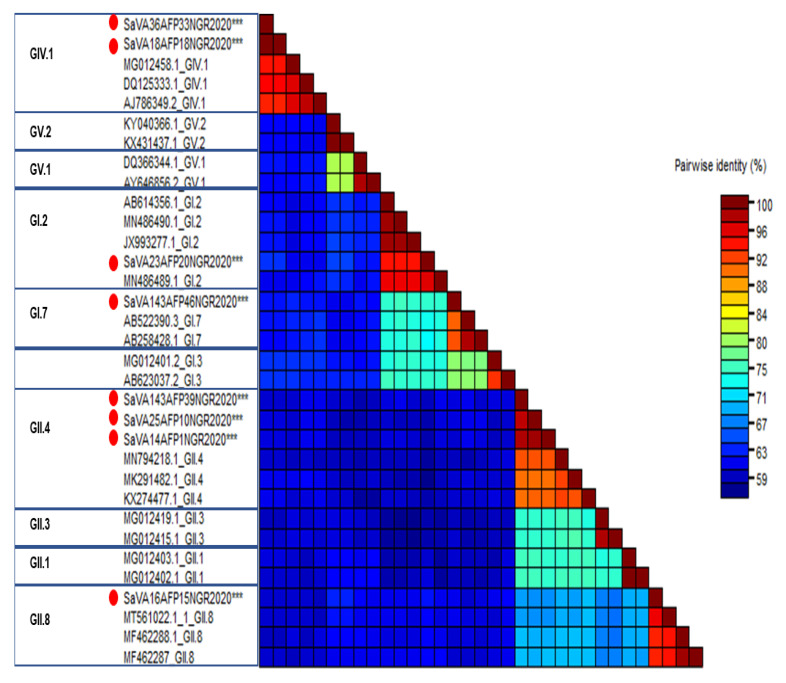
The Sequence Demarcation Tool [53] was used to estimate the pairwise sequence identity between the VP1 of SaV sequences from this study and existing SaV references. The sequences reported in this study are indicated with a red circle and astericks (***).

Eight (14.5%) of the fifty-five non-polio AFP sample pools tested using deep sequencing contained SaV reads, corresponding to 0.1% to 2.41% of the generated reads (Table 1). Of the eight samples with SaV reads, two sample pools each from Edo, Abuja, Kaduna, and Lagos states had SaV reads (Supplementary Table S1 and Figure S1). No SaV reads were detected in sample pools from the Anambra state. We obtained six complete genome sequences (SaV-A14-AFP1-NGR-2020, SaV-A16-AFP15-NGR-2020, SaV-A18-AFP18-NGR-2020, SaV-A36-AFP33-NGR-2020, SaV-A143-AFP39-NGR-2020, SaV-A143-AFP46-NGR-2020) having complete coding regions, and two partial genome sequences (SaV-A23-AFP20-NGR-2020 and SaV-A25-AFP10-NGR-2020) having VP1 and VP2 capsid gene but only partial nonstructural genes. Complete VP1-based sequence genotyping using the calicivirus typing tool and Sequence Demarcation Tool showed that the eight contigs belonged to three of the four recognized human SaV genogroups (GI, GII, and GIV). Furthermore, they belonged to genotypes GI.2 [*n* = 1], GI.7 [*n* = 1], and GII.4 [*n* = 3], and the newly discovered genotypes GII.8 [*n* = 1] and GIV.1 [*n* = 2] (Table 1, Figure 1).

Several calicivirus proteins possess conserved motifs and domains responsible for their function. Previously described conserved amino acid motifs in caliciviruses include NS3 [NTpase] (GAPGIGKT), NS5 [Viral genome-linked protein (VPg)] (KGKTK and DDEYDE), protease (GDCG), RNA-dependent RNA-polymerase (WKGL, KDEL, DYSKWDST, GLPSG, and YGDD) and VP1(PPG and GWS have been suggested to be vital in stabilizing P-domain formation in the SaV capsid) [1,18,56]. An analysis of the conserved amino acid motifs of SaV sequences in this study showed minor variations in NS3 and NS5 motifs. In the NS3, all the sequences, irrespective of the genogroup, had a GPPGIGKT motif (the A replaced with P), while in the NS5, the KGKTK motif was present in all the sequences except two (SaV-A14-AFP1-NGR-2020 and SaV-A143-AFP46-NGR-2020) that had the KGKNK motif (Table 2).

**Figure 2:**
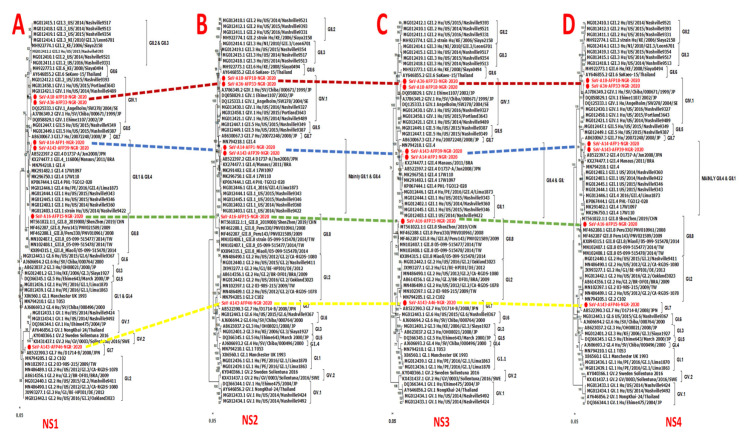
Maximum-likelihood trees of the NS1-4 genes. The trees were constructed based on the full-length amino acid sequences of (**A**) the p11 (NS1) protein, (**B**) the p28 (NS2) protein, (**C**) the NTpase (NS3), and (**D**) the p32 (NS4). Bootstrap support values greater than 50 are shown. Sequences reported in this study are highlighted in red.

**Figure 3:**
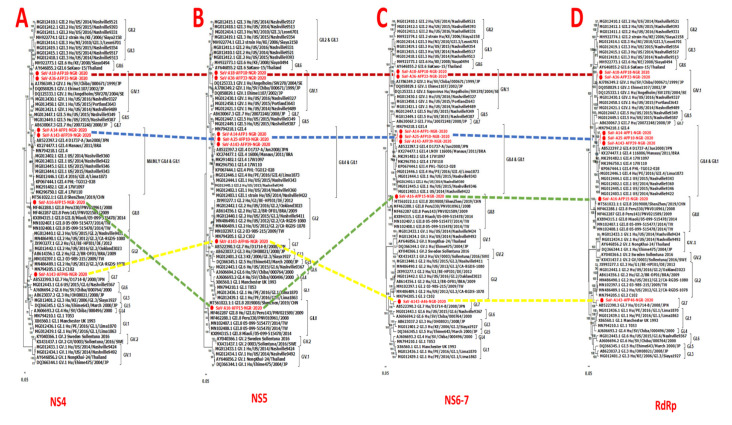
Maximum-likelihood trees of the NS4-7 and RdRp gene. The trees were constructed based on the full-length amino acid sequences of (**A**) the p32 (NS4) protein, (**B**) the viral genome-linked protein (NS5), (**C**) protease–polymerase (NS6-7), and (**D**) RdRp. Bootstrap support values greater than 50 are shown. Sequences reported in this study are highlighted in red.

**Figure 4:**
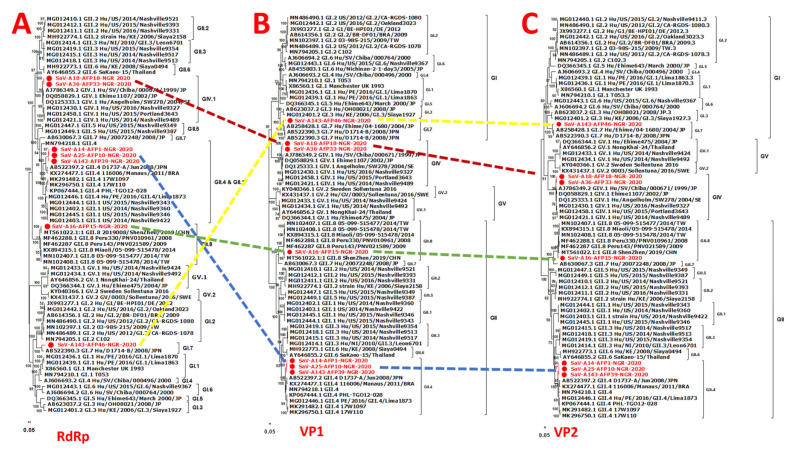
Maximum-likelihood trees of SaV genes. The trees were constructed based on the full-length amino acid sequences of (**A**) RdRp, (**B**) the capsid protein (VP1), and (**C**) the small basic protein (VP2). Bootstrap support values greater than 50 are shown. Sequences reported in this study are highlighted in red.

Phylogenetic analysis using the individual genes encoding both the structural (VP1 and VP2) and nonstructural proteins (NS1-7) and reference human SaVs showed topological incongruence. Specifically, all the nonstructural genes (Figure 2 and Figure 3) of genomes reported in this study and previously reported reference sequences, including the RdRp gene, were clustered into three main genogroups (GI, GII, and GV) (Figure 2A–D and Figure 3A–D). In contrast, the structural genes (VP1 and VP2) were grouped into four clusters (GI, GII, GIV, and GV) (Figure 4B,C). All GIV nonstructural genes were consistently found among the GII clusters, while their structural genes were in a group independent of GII. Interestingly, the GIV sequences in this study clustered independently from previously documented strains from Asia and North America. The GII.8 detected in this study clustered with a novel variant of the GII.8 genotype, which was associated with an outbreak of SaV among primary school students in Shenzhen city, China, in 2019 [57].

The human SaV sequences detected in this study did not contain any significant recombination breakpoints according to RDP4 sequence analysis.

## 4. Discussion

Without a doubt, the global health community has made significant investments and taken targeted actions to address the primary causes of child death through high-impact interventions, such as access to nutrition, safe water, sanitation, and vaccination. Malnutrition and diarrheal diseases, on the other hand, continue to be among the top causes of death among children [58,59]. In Nigeria, there is a dearth of information on SaV’s genetic diversity, epidemiology, and evolution [60]. In the present study, we describe six complete genome sequences (all with complete coding regions) and two partial genome sequences from children with AFP. This is the first detection of human SaVs in Nigeria. Interestingly, multiple genotypes were detected, indicating the circulation of various strains in Nigeria. Specifically, we documented the presence of genogroups GII (GII.4 and GII.8), GIV (GIV.1), and GI (GI.2 and GI.7) in Nigeria.

All the identified human SaVs, irrespective of genotype, had amino acid substitution A482P in the NS3 motifs (Table 2). A similar motif was reported in SaVs from pigs [25]. Since many caliciviruses, including SaVs, are difficult to grow in cell cultures, studying the biological function of their nonstructural proteins remains challenging. However, few studies have elucidated the role and activities of the polymerase and protease (3C-like protease (NS6) and the 3CD-like protease–polymerase (NS6-7) [18,61,62,63]. Interestingly, the mutational analysis of the RdRp-conserved GDD amino acid motif from a calicivirus *rabbit hemorrhagic disease virus* (RHDV) showed that the substitution of the RHDV 3D^pol^ 1605 aspartate residue by asparagine, glycine or glutamate residues resulted in a complete loss of enzymatic activity [64]. Understanding the biological functions of various SaV proteins and the role of various amino acid substitutions in the evolution of viruses is needed to understand the potential implications of newly observed mutations.

Regarding seasonality, Nigeria has two seasons in a year as follows: the wet (April to October) and dry (November to March) seasons. In this study, SaVs were detected in sample pools collected in both the wet and dry seasons, with 62.5% (5/8) of the SaVs detected in samples collected during the wet season. Our findings support the widely accepted hypothesis that SaV is primarily found in the winter and during the rainy season [8,36]. Since no other work on SaV’s prevalence on a monthly basis has been reported in Nigeria, it is difficult to determine the true prevalence of SaV in Nigeria using this approach. We are aware that the SaV’s diversity described in this study might not completely capture variants present in this sample, considering the study design. Therefore, future studies aimed at identifying the seasonal nature of SaV transmission patterns could help with infection prevention, control, and diagnosis strategies.

The genetic analysis of currently circulating SaV strains is critical for understanding the cryptic geographic distribution of SaVs in the population, both regionally and globally. Previous studies have revealed that GI is the most common SaV genogroup around the globe and has been increasingly detected in many African countries [8,39,65,66]. In this study, we observed the circulation of a variety of SaV strains throughout the year. Sapovirus GII.4 was the predominant genotype detected and was closely followed by GIV.1. It is important to note that the presence of GII.4 sequences in this study, which have been classified among the rare SaV genotypes [32], may indicate that the SaV landscape in Africa might be changing. Furthermore, the preponderance of GII.4 sequences from this research is contrary to previous SaV studies in Africa, where GI was the most abundant SaV genotype [39,40,65]. In Thailand, a significant proportion of genotype GII.4 SaV was identified [67]. GIV, on the other hand, is a genotype that was frequently detected in developed countries around 2007 [1], as well as in Africa between 2009 and 2013 [41,42].

Phylogenetically, the Nigerian SaVs were related to previously reported SaV reference strains. While GIV sequences in this study formed small sub-clusters independent from previously documented strains from Asia and North America, the GII.8 in this study was 95.4% similar and clustered with a novel variant of the GII.8 genotype, which was associated with an outbreak of SaV among primary school students in Shenzhen city, China, in 2019 [57]. The position and length of the ORFs, VP1, and VP2, of the Nigerian GII.8 strains were identical to those of the Shenzhen strain. Remarkably, the GI.7 strain from this study was more than 90% similar to the GI.7 strains from Japan that were associated with gastroenteritis outbreaks linked to the consumption of contaminated shellfish [68]. Notably, GII.4 strains were found in samples collected in Lagos (January), Kaduna (February), and Abuja (September), whereas GIV strains were found in samples collected in the Edo state (May and August 2020). These results imply that these strains may be locally circulating in Nigeria and/or that an outbreak that was not discovered may have occurred there. The robust surveillance of SaV among AGE and non-AGE cohorts in Nigeria is needed to better understand the genotype diversity, evolution, and probable disease association of this virus in the country.

Of note, the RDP4 findings and the phylogenetic tree structure did not provide adequate support to classify any of the Nigerian SaVs as recombinant strains. However, sequences from this study and other reference sequences (including the four genogroups known to infect humans) included in the alignment all showed a phylogenetic pattern in which all nonstructural genes clustered into three major genogroups (GI, GII, and GIV). The structural genes (VP1 and VP2) were divided into the following four clusters: GI, GII, GIV, and GV. A similar topology incongruence has been reported [32], which may indicate an ancient recombination event.

Some of the limitations of our study include the fact that samples suffered more than one round of freezing and thawing, which might have affected the quantity and quality of the genomes recovered in this study. We were also unable to determine the true prevalence of SaV due to our purposive sampling strategy, which included only children with AFP.

In conclusion, we describe six complete and two partial SaV genome sequences. This is the first report on human SaVs in Nigeria. Hence, the data described here can serve as references to help develop tools to enhance the surveillance of and improve epidemiological information on SaVs in Nigeria and Africa at large, where only short-genome regions have been reported. Further, understanding the evolutionary dynamics of SaV, especially the nonstructural proteins, is vital to fully delineate the role of amino acid substitutions in SaV’s evolution and genetic diversity. This would make their nonstructural proteins desirable targets for developing therapeutics to treat human calicivirus infections.

## Figures and Tables

**Table 1:** Summary of SaV reads detected in the pooled AFP samples.

Sample ID	Deep Sequencing-Derived Reads and Sequences				Calicivirus Genotyping Tool Results	BLASTn Results		
	Total Reads	SaV Reads	SaV Reads %	Nucleotide Length	VP1 Length	Type	Accession number of the Closest Hit	Pairwise Identity (%)
AFP1-NGR	16,751,880	51,665	0.31%	7483	1677	GII.4	MN794218.1	91.29
AFP10-NGR	4,179,088	882	0.02%	4554	1677	GII.4	MN794218.1	91.17
AFP15-NGR	23,759,398	24,255	0.1%	7502	1667	GII.8	MT561022.1	95.36
AFP18-NGR	7,378,192	1777	0.02%	7456	1656	GIV.1	DQ125333.1	94.27
AFP20-NGR	10,079,418	53	0%	1819	1439	GI.2	MN486489.1	97.08
AFP33-NGR	11,500,814	83,736	0.73%	7494	1656	GIV.1	DQ104357.2	94.43
AFP39-NGR	13,103,440	41,026	0.31%	7526	1676	GII.4	AB429084.2	91.06
AFP46-NGR	22,571,328	544,044	2.41%	7524	1698	GI.7	AB258428.1	90.76

Abbreviations: Nigeria, NGR; Acute Flaccid Paralysis, AFP; Sapovirus, SaV.; pairwise nucleotide identity with the top matching reference sequences for the VP1 region using BLASTn.

**Table 2:** Typical motifs of functional proteins of SaV detected in the pooled AFP samples.

Strain	NTpase GAPGIGKT	VPg (KGKTK And DDEYDE	Protease (GDCG)	RdRp (WKGL, KDEL, DYSKWDST, GLPSG and YGDD)	VP1 (PPG and GWS)
SaV-A14-AFP1-NGR-2020	^481^GPPGIGKT	^936^KGKNK And ^953^DDEYDE	^1169^GDCG	^1213^WKGL, ^1374^KDEL, ^1449^DYSKWDST, ^1504^GLPSG and ^1552^YGDD	^1856^PPG and ^2000^GWS
SaV-A16-AFP15-NGR-2020	^465^GPPGIGKT	^927^KGKTK And ^948^DDEYDE	^1154^GDCG	^1198^WKGL, ^1359^KDEL, ^1434^DYSKWDST, ^1489^GLPSG and ^1537^YGDD	^1841^PPG and ^1987^GWS
SaV-A18-AFP18-NGR-2020	^481^GPPGIGKT	^942^KGKTK And ^963^DDEYDE	^1169^GDCG	^1213^WKGL, ^1374^KDEL, ^1449^DYSKWDST, ^1504^GLPSG and ^1552^YGDD	^1851^PPG and ^1996^GWS
SaV-A36-AFP33-NGR-2020	^481^GPPGIGKT	^942^KGKTK And ^963^DDEYDE	^1169^GDCG	^1213^WKGL, ^1374^KDEL, ^1449^DYSKWDST, ^1504^GLPSG and ^1552^YGDD	^1851^PPG and ^1996^GWS
SaV-A143-AFP39-NGR-2020	^481^GPPGIGKT	^942^KGKTK And ^963^DDEYDE	^1169^GDCG	^1213^WKGL, ^1374^KDEL, ^1449^DYSKWDST, ^1504^GLPSG and ^1552^YGDD	^1856^PPG and ^2001^GWS
SaV-A143-AFP46-NGR-2020	^479^GPPGIGKT	^940^KGKNK And ^961^DDEYDE	^1167^GDCG	^1211^WKGL, ^1373^KDEL, ^1448^DYSKWDST, ^1503^GLPSG and ^1552^YGDD	^1855^PPG and ^2000^GWS

The substitution observed in NS3 and NS5 motifs are highlighted in red box.

## Data Availability

The original data presented in the study including the Sapovirus nucleotide sequences are openly available in GenBank under the accession number OR837774-OR837781. Raw sequence data from this study have also been uploaded the NCBI database under the Bioproject number PRJNA1043841.

## Supplementary Materials

The following supporting information can be downloaded at: https://www.mdpi.com/article/10.3390/pathogens13030264/s1, Table S1: Summary of samples analysed in this study including the number of samples per pool, location where samples were collected and month of sample collection; Figure S1: Map of Nigeria indicating the states (highlighted in light blue) spread across five geopolitical zones from where samples analyzed in this study were collected and the number of samples with SaV reads detected (highlighted in red diamond) from each state.
