# Supplementary material for: Metagenomic Detection and Genetic Characterization of Human Sapoviruses among Children with Acute Flaccid Paralysis in Nigeria
Source: Pathogens. 2024 Mar 19;13(3):264. doi: 10.3390/pathogens13030264 (PMC10976229; doi:10.3390/pathogens13030264)
Supplement: Supplementary file 1 [file pathogens-13-00264-s001.zip › pathogens-2857270-supplementary.pdf]

Supplementary Table 1: Summary of samples analysed in this study including the number of samples per pool, location where samples were collected and month of sample collection

Sample ID	Number of samples per pool	Location	Month
AFP01-NGR*	5	LAGOS	JANUARY
AFP02-NGR	6	ANAMBRA	JANUARY
AFP03-NGR	5	EDO	JANUARY
AFP04-NGR	5	FCT	JANUARY
AFP05-NGR	5	KADUNA	JANUARY
AFP06-NGR	5	LAGOS	FEBRUARY
AFP07-NGR	5	ANAMBRA	FEBRUARY
AFP08-NGR	5	EDO	FEBRUARY
AFP09-NGR	5	FCT	FEBRUARY
AFP010-NGR*	5	KADUNA	FEBRUARY
AFP011-NGR	2	FCT, ABUJA	MARCH
AFP012-NGR	1	KADUNA	MARCH
AFP013-NGR	5	LAGOS	APRIL
AFP014-NGR	7	EDO	APRIL
AFP015-NGR*	7	FCT	APRIL
AFP016-NGR	5	KADUNA	APRIL
AFP017-NGR	5	ANAMBRA	MAY
AFP018-NGR*	5	EDO	MAY
AFP019-NGR	4	FCT	MAY
AFP020-NGR*	5	KADUNA	MAY
AFP021-NGR	5	LAGOS	JUNE
AFP022-NGR	5	ANAMBRA	JUNE
AFP023-NGR	5	EDO	JUNE
AFP024-NGR	5	FCT	JUNE
AFP025-NGR	5	KADUNA	JUNE
AFP026-NGR	5	LAGOS	JULY
AFP027-NGR	5	ANAMBRA	JULY
AFP028-NGR	5	EDO	JULY
AFP029-NGR	3	FCT	JULY
AFP030-NGR	5	KADUNA	JULY
AFP031-NGR	5	LAGOS	AUGUST
AFP032-NGR	5	ANAMBRA	AUGUST
AFP033-NGR*	5	EDO	AUGUST
AFP034-NGR	4	FCT	AUGUST
AFP035-NGR	5	KADUNA	AUGUST
AFP036-NGR	4	LAGOS	SEPTEMBER
AFP037-NGR	5	ANAMBRA	SEPTEMBER
AFP038-NGR	5	EDO	SEPTEMBER

AFP039-NGR*	5	FCT	SEPTEMBER
AFP040-NGR	4	KADUNA	SEPTEMBER
AFP041-NGR	4	LAGOS	OCTOBER
AFP042-NGR	4	ANAMBRA	OCTOBER
AFP043-NGR	4	EDO	OCTOBER
AFP044-NGR	5	FCT	OCTOBER
AFP045-NGR	2	KADUNA	OCTOBER
AFP046-NGR*	5	LAGOS	NOVEMBER
AFP047-NGR	2	ANAMBRA	NOVEMBER
AFP048-NGR	5	EDO	NOVEMBER
AFP049-NGR	5	FCT	NOVEMBER
AFP050-NGR	5	KADUNA	NOVEMBER
AFP051-NGR	5	LAGOS	DECEMBER
AFP052-NGR	5	ANAMBRA	DECEMBER
AFP053-NGR	3	EDO	DECEMBER
AFP054-NGR	3	FCT	DECEMBER
AFP055-NGR	5	KADUNA	DECEMBER

*Sample pools from where SaVs described in the study were detected.

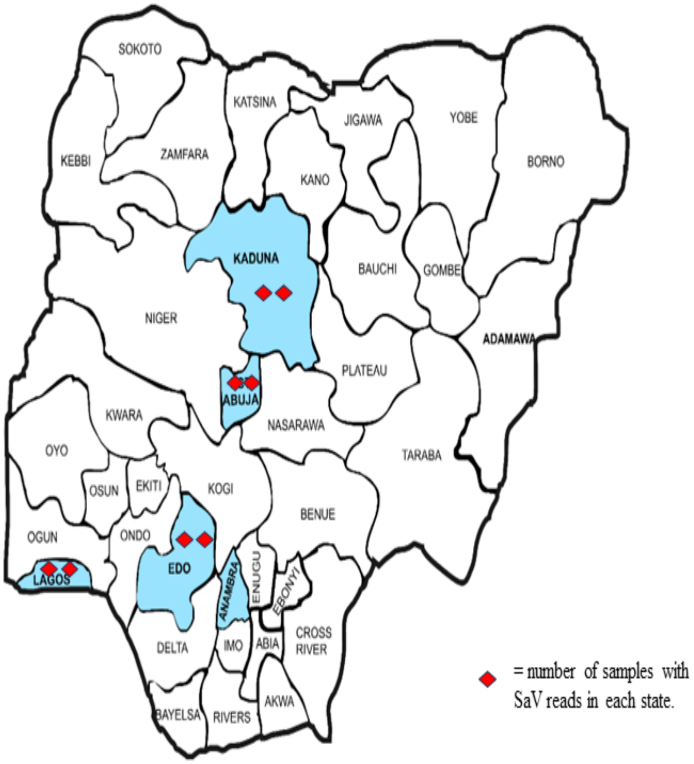

Supplementary Figure 1: Map of Nigeria indicating the states (highlighted in light blue) spread across five geopolitical zones from where samples analyzed in this study were collected and the number of samples with SaV reads detected (highlighted in red diamond) from each state.
